# Modulators of mercury risk to wildlife and humans in the context of rapid global change

**DOI:** 10.1007/s13280-017-1011-x

**Published:** 2018-01-31

**Authors:** Collin A. Eagles-Smith, Ellen K. Silbergeld, Niladri Basu, Paco Bustamante, Fernando Diaz-Barriga, William A. Hopkins, Karen A. Kidd, Jennifer F. Nyland

**Affiliations:** 1 US Geological Survey, 3200 SW Jefferson Way, Corvallis, OR 97331 USA; 2 Johns Hopkin Bloomberg School of Public Health, 615 N. Wolfe Street, E6644, Baltimore, MD 21205 USA; 3 McGill University, 204-CINE Building, Montreal, QC H9X 3V9 Canada; 4 University of La Rochelle, laboratory of Littoral Environment and Societies, Littoral Environnement et Sociétés (LIENSs), LIENSs UMR 7266 CNRS-Université de La Rochelle, 2 rue Olympe de Gouges, 17000 La Rochelle, France; 5 Center for Applied Research in Environment and Health at, Universidad Autonoma de San Luis Potosi, Avenida Venustiano Carranza No. 2405, Col Lomas los Filtros Código Postal, 78214 San Luis Potosí, SLP Mexico; 6 Department of Fish and Wildlife Conservation, 310 West Campus Drive Virginia Tech, Cheatham Hall, Room 106 (MC 0321), Blacksburg, VA 24061 USA; 7 Department of Biology & School of Geography and Earth Sciences, McMaster University, 1280 Main Street W., Hamilton, ON L8S 4K1 Canada; 8 Department of Biological Sciences, 1101 Camden Ave, Salisbury, MD 21801 USA

**Keywords:** ASGM, Biomagnification, Fisheries, Immunotoxicity, Invasive species, Microbiome, Selenium

## Abstract

Environmental mercury (Hg) contamination is an urgent global health threat. The complexity of Hg in the environment can hinder accurate determination of ecological and human health risks, particularly within the context of the rapid global changes that are altering many ecological processes, socioeconomic patterns, and other factors like infectious disease incidence, which can affect Hg exposures and health outcomes. However, the success of global Hg-reduction efforts depends on accurate assessments of their effectiveness in reducing health risks. In this paper, we examine the role that key extrinsic and intrinsic drivers play on several aspects of Hg risk to humans and organisms in the environment. We do so within three key domains of ecological and human health risk. First, we examine how extrinsic global change drivers influence pathways of Hg bioaccumulation and biomagnification through food webs. Next, we describe how extrinsic socioeconomic drivers at a global scale, and intrinsic individual-level drivers, influence human Hg exposure. Finally, we address how the adverse health effects of Hg in humans and wildlife are modulated by a range of extrinsic and intrinsic drivers within the context of rapid global change. Incorporating components of these three domains into research and monitoring will facilitate a more holistic understanding of how ecological and societal drivers interact to influence Hg health risks.

## Introduction

The health effects of mercury (Hg) exposure represent a significant threat to ecosystems and human welfare worldwide (Driscoll et al. 2013), but understanding the risks associated with Hg exposure is complicated by this element’s varied environmental fate and the overarching influences of environmental, biological, and socioeconomic drivers. In its various forms, Hg causes immunotoxicity (Hawley et al. 2009; Lewis et al. 2013; Hui et al. 2016; Zhang et al. 2016; Crowe et al. 2017) and nephrotoxicity (Tchounwou et al. 2003), diminishes neurological capacity and neurobehavioral function (Steuerwald et al. 2000; Basu et al. 2005; Clarkson and Magos 2006; Mergler et al. 2007; Scheuhammer and Sandheinrich 2008; Depew et al. 2012a; Bridges et al. 2016; Landler et al. 2017), alters functioning of three major endocrine axes (Wada et al. 2009; Meyer et al. 2014), and impairs reproduction and alters offspring quality (Klaper et al. 2006; Burgess and Meyer 2008; Bergeron et al. 2011; Hopkins et al. 2013; Tartu et al. 2013; Thompson et al. in press). The numerous adverse health effects of Hg, coupled with the diverse environmental sources, propensity for long-range transport, complex biogeochemical cycling, and various exposure pathways through food webs and from industrial activities present significant challenges to characterizing and managing the risk of Hg exposure to ecological and public health. Nonetheless, substantial progress has been made over the past several decades, resulting in improved inventories of Hg sources and releases (Streets et al. 2017), and a more robust scientific understanding of the factors influencing Hg fate and transport (see companion paper by Obrist et al. (2018), the processes driving methylmercury (MeHg) production (see companion paper by Hsu-Kim et al. (2018), and the manifestations and mechanisms of many of the toxic effects of Hg in biota. This progress has supported global efforts to reduce Hg loading to the environment for protection of human and ecological health, such as those put forth at the Minamata Convention (see companion paper by Selin et al. (2018).

Ecological and public health risks posed by Hg rarely follow a simple linear relationship from Hg releases, to uptake and exposure, to adverse health outcomes. Instead, a diverse suite of extrinsic ecological and socioeconomic factors (herein called “extrinsic drivers,” e.g., invasive species, culture) and intrinsic biological factors (herein called “intrinsic drivers,” e.g., genetics, microbiome) alter the availability of Hg for biological uptake, and modify Hg toxicity to humans and other animals. Some of these drivers—e.g., climate change, land use changes—are occurring at unprecedented global rates and challenge the development of effective Hg control actions, alter recovery trajectories, and confound interpretations of monitoring outcomes. The growing extent and intensity of some drivers will continue to influence key processes associated with Hg exposure and bioaccumulation in humans and other animals; thus, there is a critical need to understand how these drivers alter mechanisms of Hg bioavailability in order to better predict changes in Hg exposure trajectories and mitigate adverse health effects.

Exposure to MeHg mainly occurs via the diet for most organisms (Mergler et al. 2007; Wiener 2013), whereas inorganic Hg (elemental (Hg^0^) and ionic Hg (Hg^II^)) exposures (particularly in humans) largely occur through inhalation, dermal, or oral routes (Clarkson and Magos 2006), which are primarily associated with industrial processes, such as artisanal and small-scale gold mining (ASGM) (Steckling et al. 2011; Basu et al. 2015b), and medical uses (Clarkson and Magos 2006). Exposures to both organic and inorganic Hg species are influenced by the environmental alterations and societal shifts associated with extrinsic drivers (such as hydrologic and land-use alterations, invasive species, climate change, and macroeconomic change), but through very different mechanisms. For example, extrinsic drivers that alter trophic processes, e.g., food web structure, foraging ecology, and ecosystem energetics (i.e., energy flow through food webs), influence the magnitude of MeHg bioaccumulation and biomagnification through food webs (Vander Zanden and Rasmussen 1996; Lavoie et al. 2013; Karimi et al. 2016a; Polito et al. 2016). Conversely, other extrinsic drivers, such as shifts in global socioeconomic trends, cultural patterns, and development trajectories, affect risks of inorganic Hg exposure in human populations (Swain et al. 2007). It is important to also recognize the critical roles of abiotic Hg fate and transport and MeHg production in the Hg cycle, as well as their contributions to Hg exposure and health risks. Although we do not address those processes here, as they are reviewed in detail elsewhere (Hsu-Kim et al. 2018; Obrist et al. 2018), their exclusion herein should be interpreted not as diminished importance, but as an alternate focus. Instead, we examine extrinsic and intrinsic drivers that influence the routes of Hg exposure, and subsequently alter risks of Hg to human health and the health of organisms in the environment. The growing extent and intensity of some drivers will continue to influence key mechanisms associated with Hg exposure and bioaccumulation in both humans and other animals; thus, there is a critical need to understand these interactions to better predict changes in exposure trajectories and realized adverse health effects.

**Fig. 1:**
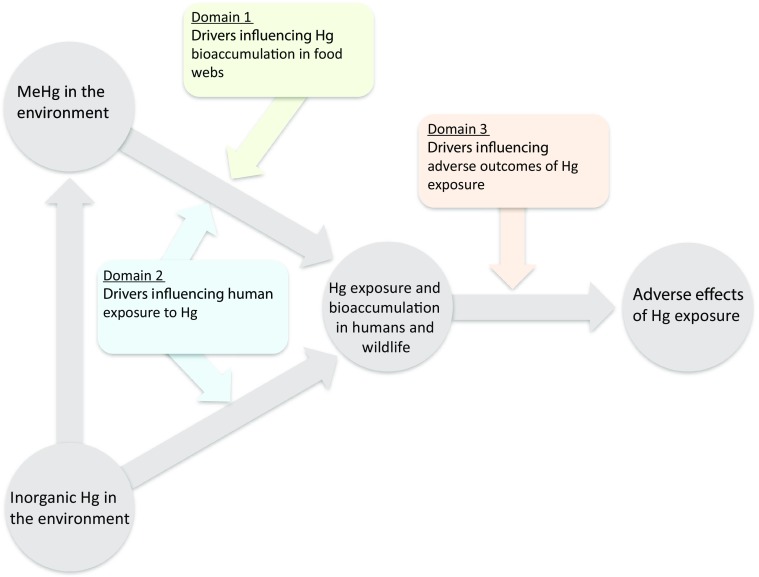
Conceptual model of mercury (Hg) pathways through the ecosphere, and the domains of drivers that influence the risk of Hg exposure and related adverse health effects. The gray arrows represent the combined physical, chemical, and biological processes that influence movement from one category to another, and the domains represent key drivers that interact with those processes to influence outcomes in the receiving categories. Global extrinsic drivers affecting methylmercury (MeHg) bioaccumulation in Domain 1 include hydrologic alteration, land-use change, invasive species, and climate change (see Fig. 2). Global extrinsic drivers that influence human exposure to inorganic Hg and MeHg in Domain 2 include changes in socioeconomic aspects of subsistence and luxury fish consumption and ASGM, whereas individual- and molecular-level intrinsic drivers within this domain include genetic variability and gastrointestinal assimilation (see Fig. 3). Extrinsic and intrinsic drivers modulating adverse health outcomes of MeHg and inorganic Hg exposure in humans and other animals in Domain 3 include exposure to pathogens and infectious disease, and variations in nutrients and co-contaminants, the microbiome, and genetics

In this paper, we examine how specific extrinsic and intrinsic drivers that operate across multiple scales of organization (global, individual, and molecular) modulate mechanisms of Hg bioaccumulation in organisms and biomagnification through food webs, Hg exposure in humans, and Hg toxicity and health outcomes. Our goal is to integrate across disciplines that commonly are not linked, but nevertheless can exert cumulative influence on Hg health risks, in order to encourage scientific development of a more unified strategy for quantifying Hg risk during a time of rapid global change. As such, our objectives are to (1) examine the relationship between global change drivers and Hg bioaccumulation and biomagnification through ecosystems, (2) discuss key factors that influence human exposure to MeHg and inorganic Hg, and (3) summarize interactive factors that can modulate toxic responses to Hg in wildlife and humans. This approach explicitly incorporates ecological, socioeconomic, and physiological factors to assist with quantifying the effectiveness of Hg-reduction efforts on health outcomes and ecological risk. We address these objectives within a conceptual framework based upon pathways of Hg cycling through the ecosphere (Fig. 1), and within three key **domains** of ecological and human health risk. **Domain 1** represents the *extrinsic global change drivers that influence MeHg bioaccumulation and biomagnification through food webs*. **Domain 2** describes the global *extrinsic drivers, and individual*- *and molecular*-*level intrinsic drivers that influence human exposure to both MeHg and inorganic Hg*, as well as how these drivers interact with those of **Domain 1** to modulate Hg exposure through the human consumption of Hg-contaminated foods. **Domain 3** comprises the *extrinsic and intrinsic drivers across a range of scales that influence the adverse effects of both MeHg and inorganic Hg in human and ecological health endpoints*, and how they interact with the global change drivers described in Domains 1 and 2.

## Domain 1: Extrinsic global change drivers that influence METHYLMERCURY bioaccumulation and biomagnification in food webs

The unprecedented rate of human-induced changes in hydrology, land use, invasive species, and climate is altering ecosystem processes on a global scale, including the distributions and interactions of species across terrestrial, freshwater, and marine habitats (Vitousek 1994; Hooper et al. 2012). Ecosystems respond to these extrinsic drivers in many ways, including through changes in food web structure and ecosystem energetics (Petchey et al. 1999; Baxter et al. 2004; Woodward et al. 2010), which influence MeHg bioaccumulation in food webs and subsequent dietary exposure to humans.

**Fig. 2:**
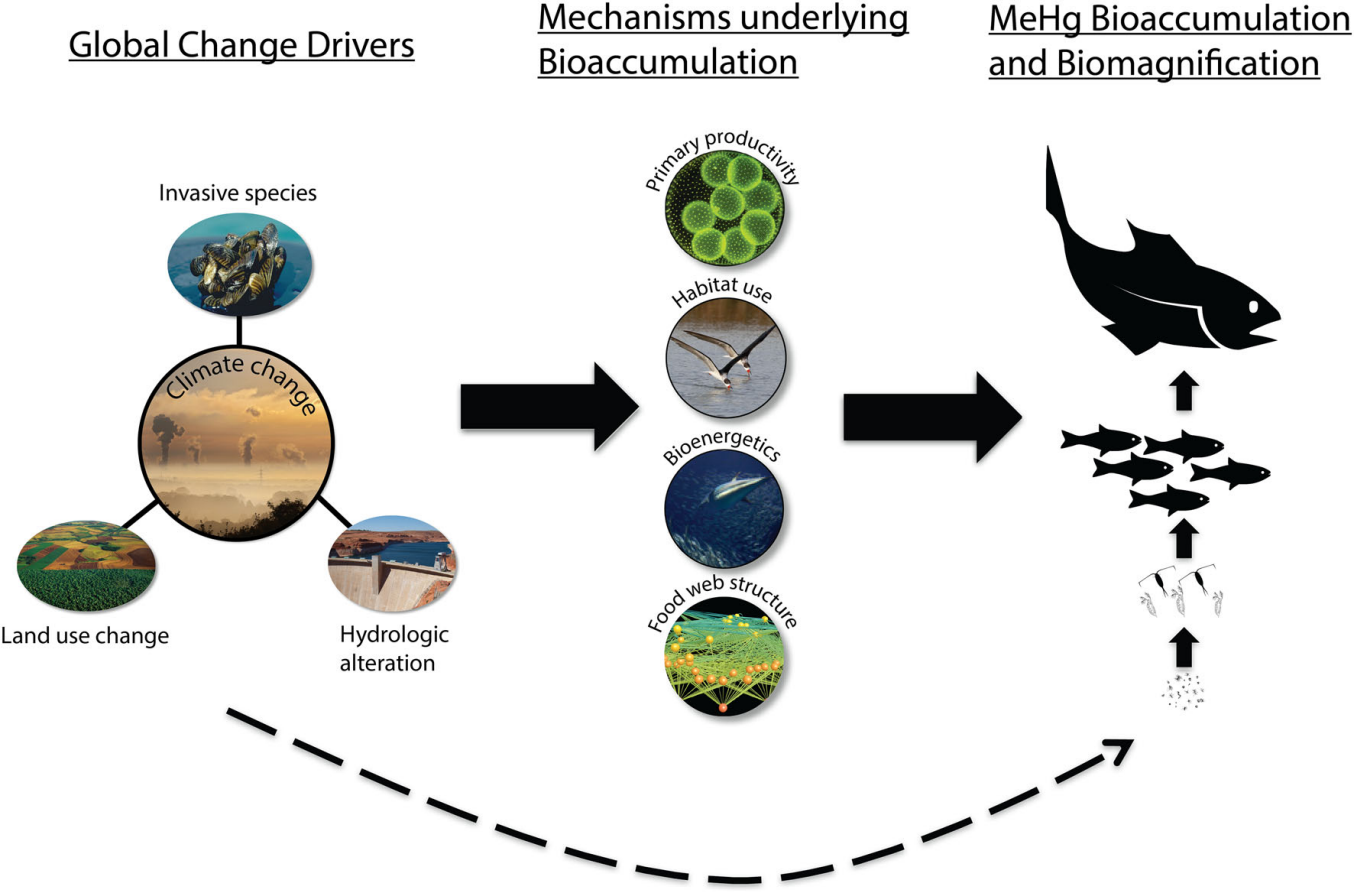
Major extrinsic drivers of global change (hydrologic alteration, land use change, invasive species, and climate change) indirectly (dashed arrow) influence methylmercury (MeHg) bioaccumulation and biomagnification through their direct effects (solid arrow) on key ecological mechanisms underlying MeHg bioaccumulation (primary productivity, habitat use, bioenergetics, and food web structure)

Herein, we identify four key ecological mechanisms that underlie changes in MeHg bioaccumulation through their effects on MeHg entry at the base of the food web, trophic transfer and biomagnification of MeHg through food webs, and toxicokinetics of MeHg within organisms. These mechanisms include (1) primary productivity, (2) habitat use, (3) bioenergetics, and (4) food web structure. Although there are some inherent overlaps among these mechanisms (e.g., hydrology dictates the available habitat, which can alter food web structure, etc.), their unique influences on MeHg bioaccumulation, biomagnification, and exposure warrant separate discussions. We then examine how each of these mechanisms can modulate (confound, moderate, or mediate) MeHg bioaccumulation and biomagnification when influenced by widespread global change drivers (Fig. 2). The extrinsic drivers considered in this domain are (1) hydrologic alteration, (2) land-use change (and associated changes to nitrogen cycling), and (3) invasive species. In addition, each of these extrinsic drivers occur within the context of (4) climate change, which modulates many aspects of the other drivers of global change. For each extrinsic driver presented below, we discuss its respective influences on one of the four mechanisms of bioaccumulation drawing support from the literature.

### Hydrologic alteration

Hydrologic alteration is a defining characteristic of the Anthropocene and a major component of global change (Rosenberg et al. 2000). Dam, levee, and canal construction, wetland draining, and restriction of tidal exchange have fundamentally transformed the global water cycle, altering the ecology of freshwater ecosystems (Sahagian 2000), adjacent terrestrial riparian habitats (Poff et al. 2007), and receiving estuaries (Herbert et al. 2015). Globally, approximately 800,000 dams regulate over half of the world’s river systems (Friedl and Wuest 2002; Nilsson 2005). These dams obstruct an estimated two-thirds of freshwater flowing to the oceans (Nilsson and Berggren 2000), and redirect approximately 50% of the planet’s accessible freshwater runoff for human use (Postel et al. 1996). Although dam construction has slowed since the 1970s, human population growth and economic development continue to drive a nearly exponential increase in global water use (Vorosmarty and Sahagian 2000). As a result, ongoing management of hydrology through reservoir operations, wetland restoration and management, and agricultural use maintains the global extent of continued hydrologic alteration, particularly in developing regions of the world (Janse et al. 2015). In particular, recent inventories suggest that small ponds created for agriculture and livestock purposes represent a substantial, previously unappreciated, and growing component of freshwater habitats (Downing et al. 2006), and they are disproportionately important for biogeochemical processes and as aquatic habitats for diverse organisms (Downing 2010).

Aquatic ecosystem functions are profoundly influenced by hydrologic alteration, resulting in changes to habitat connectivity and quality, community composition, and energy flow through ecosystems (Vorosmarty et al. 2000). Dams fragment riverine habitats (Rosenberg et al. 2000), alter water temperatures, and homogenize variation in discharge (Sabo et al. 2010). These physical and chemical changes manifest as ecological outcomes such as enhanced invasion of non-native species (Havel et al. 2005), community shifts from fluvial specialists to habitat generalists (Haxton and Findlay 2009), and increased food chain length (Sabo et al. 2010). The impacts of wetland loss on biodiversity and ecological function have been thoroughly described (Zedler and Kercher 2005), but even restored wetlands that are hydrologically managed to mirror pre-disturbance features exhibit both structural and functional biological degradation (Moreno-Mateos et al. 2012). Thus, many of the effects of hydrologic alteration are directly tied to the four key mechanisms of Hg bioaccumulation: primary productivity, habitat use, bioenergetics, and food web structure. The extent and magnitude of hydrologic alteration suggests that it may have a potentially significant impact on Hg bioaccumulation and biomagnification through food webs.

Fish and wildlife exposure to MeHg inherently differs among waterbody and habitat types (Ackerman et al. 2016; Eagles-Smith et al. 2016a) because hydrology influences the biogeochemical processes associated with MeHg production, as well as habitat availability and use, food web structure, and bioenergetics. As a result, hydrologic alteration is arguably among the most well-studied global change drivers of altered MeHg bioaccumulation in aquatic food webs. Reservoirs in particular have been associated with elevated MeHg bioaccumulation. Methylmercury concentrations in reservoir fish are between 1.4 to several fold higher than concentrations in fish from natural lakes (Kamman et al. 2005; Bodaly et al. 2007; Monson et al. 2011; Willacker et al. 2016), and the differences are particularly apparent in newly inundated reservoirs where rapid post-impoundment increases in MeHg concentrations occur through aquatic food webs. Studies in Scandinavia (Porvari 1998), North America (Willacker et al. 2016), Europe (Kruzikova et al. 2011), South America (Hylander et al. 2006), and Asia (Li et al. 2013) have all shown that total Hg and MeHg concentrations in aquatic food web components increased 3- to 30-fold within a few years after reservoir creation, with the magnitude of increases tied to amount of flooded area relative to reservoir surface area (Bodaly et al. 2007). Fish MeHg concentrations reached their maximum levels 2–14 years after impoundment across 8 hydroelectric reservoirs in Canada (Bodaly et al. 2007), and an average of 3 years after impoundment across dozens of reservoirs throughout western North America (Willacker et al. 2016). Because MeHg concentrations and reproductive risk in piscivorous birds are highly correlated with MeHg concentrations in prey fish (Evers et al. 2008; Ackerman et al. 2015), waterbird species attracted to reservoirs or their discharge waters may be at particularly high risk to MeHg exposure in the years following reservoir creation. Reservoir creation can also enhance the magnitude of aquatic invertebrate emergence, increasing the available biomass of Hg-contaminated prey and associated Hg exposure in insectivorous birds (Gerrard and St Louis 2001), providing biologically mediated subsidies to adjacent terrestrial habitats. The pulse in MeHg bioaccumulation through food webs does not occur in all newly impounded reservoirs however (Li et al. 2015), and although the mechanisms are not well understood, evidence suggests that food chain length in reservoirs may be an important regulating factor (Razavi et al. 2014; Ouedraogo et al. 2015).

Mercury concentrations in reservoir fish (and likely other taxa) eventually return to near pre-impoundment levels, although it can take several decades (French et al. 1998; Bodaly et al. 2007; Willacker et al. 2016) with rates of decline ranging between 0.5 and 3.9% per year (Green et al. 2016). However, both reservoir management and hydrologic structure can confound any return to background concentrations. Water level management, specifically the magnitude of between-year changes in maximum water levels, is linearly correlated with fish Hg concentrations in subsequent years (Sorensen et al. 2005; Larson et al. 2014; Willacker et al. 2016) and has been associated with up to a threefold difference in fish Hg concentrations from reservoirs with low versus high proportional changes in maximum water levels (Willacker et al. 2016). Water column stratification also influences food web Hg bioaccumulation, both within reservoirs (Slotton et al. 1995; Perron et al. 2014), as well as downstream environments. Elevated Hg concentrations in food webs can extend up to 200 km below dams (Kasper et al. 2014).

The influence of hydrologic alteration on Hg dynamics within food webs extends to other water bodies. For example, fish Hg concentrations have been shown to be highest in wetlands with the greatest degree of water level fluctuation (Snodgrass et al. 2000; Eagles-Smith and Ackerman 2014), and white ibis chick Hg concentrations were directly tied to water level fluctuation rates in the Florida Everglades (Herring et al. 2013). Highly managed, small agricultural ponds, represent and important habitat in semi-arid regions with limited aquatic habitat availability (Chumchal and Drenner 2015), where invertebrate MeHg flux to terrestrial environments differed among ponds with varying water management and presence or absence of fish (Henderson et al. 2012; Tweedy et al. 2013). Further, river geomorphology can influence aquatic–terrestrial Hg transfer through the collective effects on emergent insect body burdens and aquatic insect community composition (Sullivan et al. 2016).

### Land-use change

Rapid and irreversible conversion of lands for the production of food, extraction of resources, and urbanization is occurring globally. These alterations have disturbed natural hydrologic, geochemical, and biological processes due to the production and discharge of wastes, impervious surfaces of cities, conversion of forests to pastures for livestock and crop monocultures, and addition of fertilizers and pesticides to increase food production (Tilman et al. 2001). Widespread changes to terrestrial systems and unsustainable resource consumption by a rapidly growing population has resulted in global shifts in the nitrogen cycle (Vitousek 1994), unprecedented losses in biodiversity (Phalan et al. 2011), pervasive eutrophication of aquatic ecosystems (Carpenter and Bennett 2011), and impacts on regional and global climate (Vitousek 1994; IPCC 2014).

These broad-scale changes to the landscape are also altering the fate of Hg through their effects on the structure and function of terrestrial and aquatic systems and the four key mechanisms governing MeHg bioaccumulation (primary productivity, habitat use, bioenergetics, and food web structure). For example, direct toxicity, changes in nutrient flow, and habitat loss can each result in losses of sensitive species and biodiversity, which affects food web structure (e.g., urban effluents (Wenger et al. 2009; Holeton et al. 2011); agricultural pesticides and fertilizers; forestry, (Richardson and Beraud 2014)). The growth rates and bioenergetics of species shift as a result of changes in food supply, competition, and predation (Brinkmann and Rasmussen 2010). Losses in forest cover and permeable lands alter the timing and magnitude of *water flow* from terrestrial to aquatic systems (Sampaio da Silva et al. 2009; Wenger et al. 2009). Finally, the primary productivity of ecosystems from heavy reliance on fertilizers is increasing the availability of limiting nutrients to terrestrial and aquatic vegetation (Vitousek et al. 1997; Carpenter et al. 1998).

Of the four key mechanisms, alterations in primary productivity and its effects on MeHg in consumers is arguably one of the better studied, but controversies remain over the resultant net effects on Hg uptake in biota. Increases in the biomass of primary producers may dilute MeHg concentrations in the base of the food web [algae (Pickhardt et al. 2002; Perron et al. 2014)] and subsequently in predators (e.g., zooplankton (Chen and Folt 2005), fish (Larsson et al. 1992; Kidd et al. 1999; Essington and Houser 2003)). Alternatively, MeHg production may be enhanced due to a greater abundance of primary producers (Lepak et al. 2015), thereby facilitating in situ Hg methylation (Mauro et al. 2002; Paranjape and Hall 2017). Increases in the primary productivity of artificial streams by several orders of magnitude led to comparable declines in MeHg concentrations of both algae and algal consumers, supporting the biodilution hypothesis (Walters et al. 2015), whereas other experimental evidence showed either no effect or increased MeHg concentrations resulting from enhanced primary production (Rudd and Turner 1983; Jones et al. 2013). In addition, a review of several case studies examining fish Hg concentrations across a gradient of nutrient loading to coastal waters found examples that both supported and challenged the biodilution hypothesis (Driscoll et al. 2012). Although contradictory, the experimental and field evidence clearly shows that primary productivity can both reduce and enhance MeHg bioaccumulation, and it is important to resolve the contexts in which each of these responses are more likely to occur. Agricultural runoff and municipal wastewaters are the largest contributors to eutrophication of freshwater and marine coastal systems, yet there are surprisingly few studies of their impacts on Hg cycling in aquatic ecosystems and in the terrestrial landscape upon which pesticides, wastewater biosolids, fertilizers, and manures are applied.

### Invasive and introduced species

Long distance transportation and globalization have resulted in the intentional and unintentional transport of microbes, flora, and fauna around the world (Pimentel et al. 2000; Richardson et al. 2000). Non-native species that become established in new localities can outcompete or prey upon native species, introduce diseases, alter critical ecosystem functions, influence water and food supplies for humans, and cause extraordinary economic losses (Mack et al. 2000; Pimentel et al. 2000; Richardson et al. 2000; Pimentel et al. 2001, 2005). As a result, invasive species are now widely recognized as one of the greatest anthropogenic challenges facing the planet (Mack et al. 2000; Pimentel et al. 2005).

Invasive invertebrates, plants, and animals can upend ecosystem-level processes, alter food web composition, and influence the health and survival of native organisms in the receiving ecosystem. For example, invasive plants can differ from native plants in their physiology and phenology, sometimes producing striking differences in biomass, primary productivity, rhizosphere activity, and fundamental alterations to nutrient and water cycles (Ehrenfeld 2003; Gentes et al. 2013). Invasive invertebrates such as zebra mussels can overwhelm aquatic systems and cause wholesale changes in phytoplankton and zooplankton community structure, driving cascading effects on the diets and growth of fish and other aquatic animals (McNickle et al. 2006). Invasive vertebrates, such as Burmese pythons, brown tree snakes, cane toads, and fish, can alter the growth rates and population densities of native vertebrates, and in extreme cases can eliminate native species altogether through predation, pathogen transmission, resource competition, toxicity, and/or competitive exclusion (Vander Zanden et al. 1999; Phillips et al. 2003; Wiles et al. 2003; Dorcas et al. 2012; Willson 2017). Importantly, the effects of invasive species on ecosystem processes and food web structure can vary based on the characteristics of the recipient ecosystem, suggesting that a variety of site-specific factors influence the ecological outcomes of species introductions (Ehrenfeld 2003; Occhipinti-Ambrogi and Savini 2003; Vander Zanden et al. 2003; Swanson et al. 2006).

Despite the profound impacts that invasive species have on ecological systems, surprisingly little is known about how they influence the dynamics of co-occurring anthropogenic pollutants such as MeHg. One mechanism by which invasive species may influence movement of MeHg through food webs is by altering hydrology, biogeochemistry, and microbial processes that control site-specific MeHg production, facilitating subsequent bioaccumulation. For example, saltcedar (*Tamarix* spp.) introductions across the western U.S. may have modified the hydrology of reservoirs, streams, and floodplains due to evapotranspiration (reviewed in Shafroth et al. (2005)). Likewise, the highly invasive common reed (*Phragmites australis*) forms dense monocultures in both freshwater and brackish systems, excluding other plant and animal species, reducing light penetration, and modifying decomposition rates and nutrient cycling (Meyerson et al. 2000). Similarly, introduced aquatic macrophytes can overwhelm aquatic habitats, particularly under eutrophic conditions. The macrophytic rhizosphere is critical to Hg methylation, and thus has the potential to increase bioavailability to local fauna (Gentes et al. 2013). Although the impact of these nonnative plant introductions on Hg accumulation in resident food webs have not been considered, these examples illustrate the broad ecological changes that invasive species can cause, and the high probability that these alterations could influence MeHg dynamics.

Relatively few studies have directly considered how invasive species influence bioaccumulation of Hg in receiving ecosystems, and most of these have focused on changes in feeding ecology and trophic structure due to invasions or introductions. Because where an organism feeds can influence MeHg exposure and subsequent MeHg bioaccumulation (Power et al. 2002; Karimi et al. 2016a), one process that invasive species could affect, that would in turn modify MeHg fate, is changes to the foraging habitat. Indeed, threadfin shad (*Dorosoma petenense*) introduced to a lake in California, USA outcompeted resident planktivorous species for zooplankton, causing resident fishes to shift from pelagic to benthic prey. The dietary shift resulted in a 50% increase in Hg concentrations of resident planktivorous fish (Eagles-Smith et al. 2008). Alternatively, introduced species can alter the length of trophic pathways and thus influence Hg dynamics within food webs. For example, introduced rainbow smelt (*Osmerus mordax*) in Canadian lakes feed at slightly higher trophic positions than native forage fish and could thus theoretically expose predatory fish to higher concentrations of dietary Hg. However, support for this hypothesis is mixed (Evans and Loftus 1987; Franzin et al. 1994; Vander Zanden and Rasmussen 1996; Hrabik et al. 1998; Johnston et al. 2003), possibly because localized water chemistry and rapid growth rates of fish (i.e., growth dilution of Hg) may offset relatively fine-scale changes in trophic position (Swanson et al. 2006).

In light of the diverse effects that introduced species can have on ecological systems and their widespread and growing presence, considerable research is needed to understand how invasive species influence MeHg bioaccumulation. The examples above suggest this area continues to emerge as a key aspect of the overlap between invasive species and MeHg bioaccumulation through food webs. More data are needed to understand the conditions under which invasive species are most likely to influence MeHg bioaccumulation in ecosystems by altering methylation processes, food web structure, feeding ecology, hydrology, and bioenergetics of resident communities. Baseline tissue Hg concentrations will be essential to elucidate pre- and post-invasion changes to MeHg dynamics, and studies will need to consider site-specific ecological factors that may influence MeHg bioaccumulation resulting from invasions.

### Climate change

Whereas hydrologic alteration, land-use change, and invasive species are examples of localized drivers occurring globally, climate change is a global phenomenon with both global and localized impacts. As a result, it both directly and indirectly influences the four key mechanisms of MeHg bioaccumulation (primary productivity, habitat use, bioenergetics, and food web structure). It directly influences each mechanism by changing the physical and chemical properties of the environment, which triggers ecological responses. It also indirectly influences each mechanism of MeHg bioaccumulation because climate change motivates adaptation in land use (Dale 1997; Gao and Liu 2011) and water storage and conveyance (Christensen et al. 2004; Olden and Naiman 2010), while also creating expanded opportunities for non-native species invasions (Rahel and Olden 2008). Thus, climate change indirectly influences MeHg bioaccumulation and biomagnification through alterations in primary productivity, and food web structure, as well as bioenergetics.

Because diet is the primary MeHg exposure route, MeHg follows energetic pathways through food webs and is tied to an organism’s energy expenditure, acquisition, and storage. The link between MeHg bioaccumulation and bioenergetics is most commonly exhibited through growth dilution, whereby Hg concentrations are lower in faster growing individuals with higher growth efficiency than those that grow more slowly and have lower growth efficiency. This process has been demonstrated in both fish (Ward et al. 2010) and birds (Ackerman et al. 2011), and is commonly visible in the negative relationship between Hg concentrations and body condition (Eagles-Smith et al. 2016b; Baumann et al. 2017). Growth efficiency and associated bioenergetics can be influenced by either changes in basal metabolic rate (Dijkstra et al. 2013), activity costs, or food quality (Lepak et al. 2012; Johnson et al. 2015; Karimi et al. 2016a). Thus, environmental conditions that influence these factors, such as temperature, can also modulate MeHg bioaccumulation and biomagnification. For example, global average sea surface temperature has increased by 0.11 °C per decade between 1971 and 2010, and is projected to further increase by 1–3 °C over the next 50 years (IPCC 2014). Many freshwater systems may endure even higher temperature increases (Magnuson et al. 1997). Microcosm and experimental laboratory studies have shown that MeHg concentrations in killifish increased substantially over a 3 and 7 °C temperature gradient, respectively, and changes in concentrations were largely due to increased food consumption to maintain the higher basal metabolic rate associated with warmer water temperatures (Dijkstra et al. 2013). Metabolic allometric scaling theory predicts that higher temperatures and associated metabolic rates will also induce community shifts toward smaller body sizes, which has further trickle-down ramifications for ecosystem metabolic processes (Woodward et al. 2010). Among those relevant to MeHg bioaccumulation are changes in food quality and energy density (Ficke et al. 2007, Atcheson et al. 2012). Lower energy content in food requires higher consumption rates to meet metabolic needs. Thus, even if MeHg concentrations in prey remain unchanged, MeHg exposure in consumers would increase as they attempt to meet basal energy requirements. These processes have important implications for evaluating the effectiveness of global Hg reductions because changes in Hg concentrations of sentinel species may respond to both changes in Hg releases as well as extrinsic drivers such as climate change. As a result, strategies need to be developed that incorporate the role of these extrinsic drivers into models of MeHg bioaccumulation.

Cumulatively, the four extrinsic global change drivers described above (hydrologic alteration, land-use change, invasive species, and climate change) will continue to change patterns of MeHg bioaccumulation and biomagnification in complex ways. The contexts in which these drivers act is important to define in order to begin incorporating their effects into risk determination because their influence on MeHg bioaccumulation patterns will ultimately affect MeHg concentrations in fish, which is the primary source of MeHg to most humans.

## Domain 2: Global extrinsic and individual/molecular-level intrinsic drivers of MERCURY exposure in humans

Most human populations are exposed to Hg through the consumption of MeHg-contaminated fish, shellfish, and marine mammals (Sheehan et al. 2014), although rice has also emerged as a major MeHg vector in some populations (Zhang et al. 2010; Hsu-Kim et al. 2018). Human exposure to elemental or inorganic Hg can occur from sources present in certain occupational settings and contaminated sites as well as from Hg-containing products. As a result, approaches to minimize Hg exposure in humans encounter inherent conflicts because many of these Hg sources also have great value for public health, such as seafood consumption and the use of dental amalgams. Limiting exposure to these and other potential Hg sources can thus have negative health implications. Although alternatives to these Hg sources exist (e.g., Hg-free dental restoration options and low-Hg fish choices), they are often limited to more economically developed nations or would result in the elimination of culturally important food items. Thus, predicting the health risks associated with MeHg and inorganic Hg exposure in human populations is not only complicated by the global change drivers discussed in **Domain 1** that influence Hg concentrations in food items that humans consume, but also by global economic and societal drivers.

Intrinsic drivers such as genetic variability also influence human exposure to Hg, as well as the relationship between actual Hg exposure and the commonly accepted biomarkers that are used to estimate exposure in humans (i.e., blood Hg concentration reflects exposures to both organic and inorganic Hg, whereas Hg concentrations in hair and urine reflect exposures to organic and inorganic Hg, respectively). This is an important aspect of understanding human exposure to Hg because standard risk assessments often assume a constant and linear relationship between Hg exposure and different biomarker levels, despite evidence of tremendous variability in those relationships (Canuel et al. 2006). The potential lack of predictable concordance between MeHg or inorganic Hg exposure in humans and common biomarkers of exposure can have significant implications for the use of these surveillance and monitoring tools in risk assessments of Hg exposure, as well as epidemiological studies of the potential health effects of Hg exposure.

**Fig. 3:**
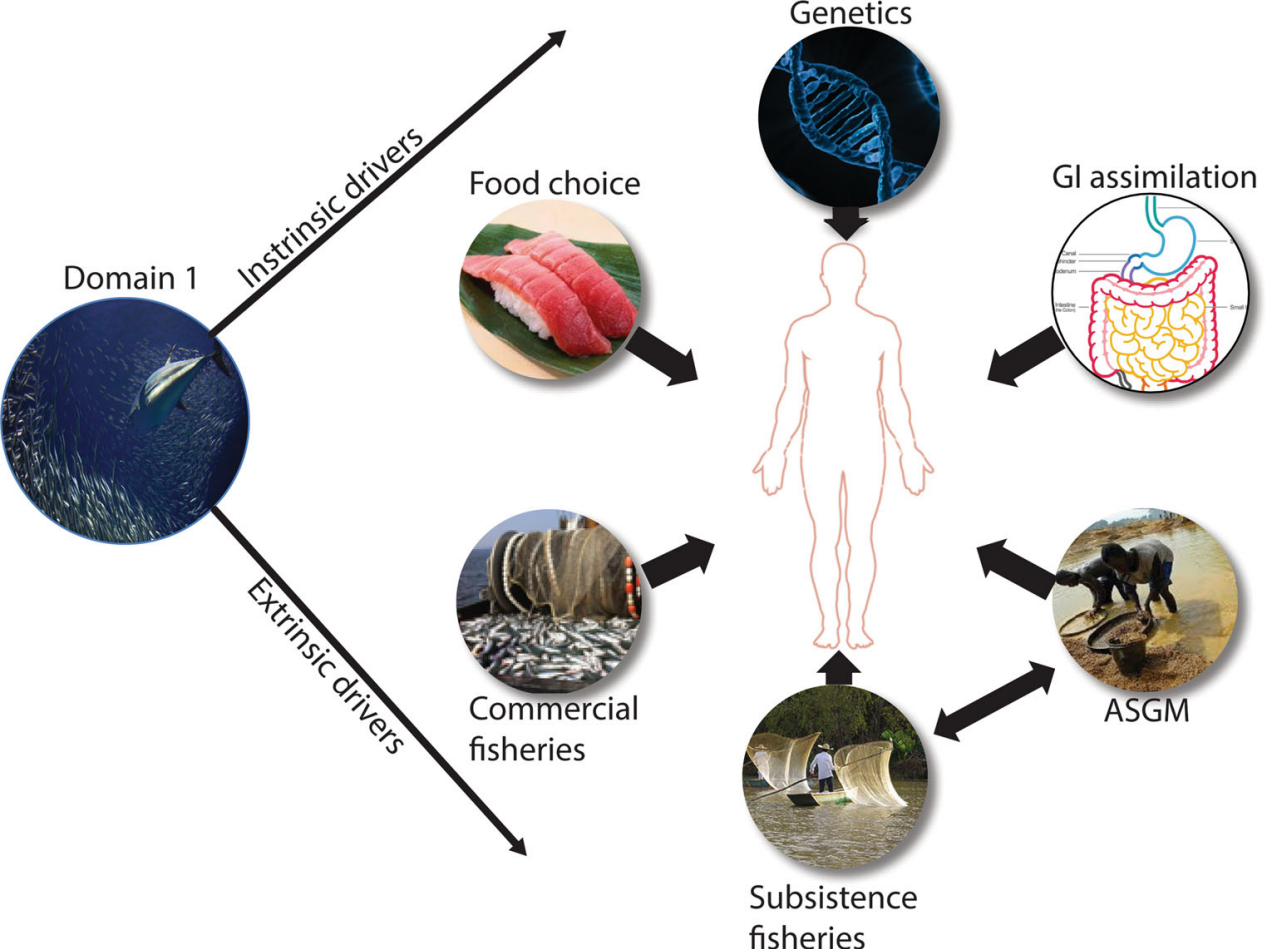
Domain 2 represents extrinsic and intrinsic drivers that influence mercury (Hg) exposure in human populations. Domain 2 is influenced by the extrinsic drivers discussed in Domain 1 as they relate to human exposure to methylmercury through the consumption of Hg-contaminated fish

In this section, we examine the key drivers associated with Hg exposure in humans that scale from individual- and molecular-level drivers, such as genetic variability, to global drivers, such as socioeconomics (Fig. 3). Specifically, we describe key socioeconomic factors that influence Hg exposure patterns in different cultures and populations as well as extrinsic and intrinsic individual-level drivers that may confound, moderate, or mediate Hg exposure and assimilation in humans.

### Global socioeconomic drivers: Extrinsic

#### Subsistence fishing, high-end economic fish, and rice consumption

Socioeconomic status is a common factor that drives disparities in Hg exposure among communities (Nriagu et al. 2012). The relationships between socioeconomic status and MeHg exposure in humans is nonlinear and is complicated by the numerous pathways of exposure. Both poverty and wealth portend Hg exposure risk for many communities, creating challenges to the development of policies to reduce risk, and to predict how risks will change with shifts in global trade and economic development. In industrialized nations, lower income urban anglers, especially minority and immigrant populations, can be at particularly high risk of Hg exposure because they are more likely to consume self-caught rather than store-bought fish, and often these anglers target predatory species with the highest Hg concentrations (Nriagu et al. 2012; Lauber et al. 2017). Risks of exposure to Hg and other environmental contaminants are also elevated for these populations because they are less likely to trust and/or adhere to fish consumption advisories (Perez et al. 2012; Niederdeppe et al. 2015). At the other end of the spectrum, individuals who more frequently consume tuna steak or swordfish, typically those in higher socioeconomic populations, are also at increased risk of elevated Hg exposures (Hightower and Moore 2003; Mahaffey et al. 2009; Karimi et al. 2014; Goodrich et al. 2016). A third group includes Indigenous populations and rural subsistence fishing communities in resource-limited regions (Chan et al. 2003). Coastal Indigenous groups represent approximately 0.4% of the global human population, but they consume an amount of fish equal to 2% of the global commercial catch (Cisneros-Montemayor et al. 2016), and their average per-capita consumption of seafood is 15 times higher than national averages (Cisneros-Montemayor et al. 2016). Some of the most impacted coastal groups are circumpolar Inuit populations, who have among the world’s highest MeHg exposures due to their particularly high reliance on marine mammals and fish consumption, although rapid changes in climate, food availability, and dietary choices are complicating risk assessments (AMAP 2015). Often, fish consumption in many of these communities not only serves as a critical source of protein, but also has a strong cultural connection, thus presenting unique social justice and risk communication complications. Encouraging these communities to consume alternative fish species with lower Hg concentrations may benefit their health, but jeopardize key aspects of their heritage, whereas maintaining traditional practices may preserve heritage, but have negative health consequences. In addition, recent evidence suggests that the majority of MeHg exposure in some rural Asian populations occurs through rice consumption (Zhang et al. 2010). The magnitude and extent of MeHg exposure risk through rice is not yet clear, but appears particularly acute in rural areas with Hg-contaminated soils (Feng et al. 2008).

As industrial development continues and economies of different countries shift in response to globalization, population growth, and resource availability, the relative Hg exposure risk in distinctive populations is likely to change in response. Similarly, continued exploitation of fisheries and “fishing down” marine food webs (Pauly et al. 1998; Essington et al. 2006; Baum and Worm 2009) is expected to influence MeHg concentrations in common market fish, thereby changing the exposure risk of the regions in which those species are commonly sold (see Box 1).

**Table:** 

**Box 1** Global change and Hg exposure through fish consumption Worldwide the most important MeHg source to human populations is the consumption of contaminated seafood. The amount of Hg that ends up in commercially available seafood depends on a range of environmental factors such as atmospheric loading rates, ecosystem-specific biogeochemical properties, and food web structure (Selin et al. 2010; Sunderland and Selin 2013), as well as socioeconomic factors such as market supply and demand. Of all seafood, Hg from tuna from the commercial market is often the most dominant source for human consumption in the U.S. (Sunderland 2007; Karimi et al. 2012), Japan (Nakagawa et al. 1997; Yamashita et al. 2005), and Mexico (Cantoral et al. 2017). Over the past few decades, the consumption of tuna sashimi has expanded from the Japanese market to a global one, contemporaneous with new distribution systems in which large supermarket and retailers are being favored over fish markets and auctions. Canned tuna production and demand continues to rise, with key markets being the U.S., the European Union, and Japan. However, as wealth increases elsewhere, these regions are also declining as a percentage of the world market. The global demand and preferences for specific fish species has also had substantial effects on the composition and structure of marine food webs (Pauly et al. 2005; Daskalov et al. 2007). In particular, global markets for upper-trophic-level species has reduced the mean trophic level and average size of fish harvested over the past several decades (Pauly et al. 1998, 2005). Simultaneously, global demand to support the growing market for fish oil and fishmeal has resulted in substantial increases in the harvest of “forage fish” species that occupy lower trophic positions (Essington et al. 2006; McClanahan et al. 2015). These harvest-induced changes to marine food webs certainly have implications for MeHg concentrations in market fish and associated human MeHg exposure, but these relationships have not been thoroughly investigated. Finally, aquaculture production has increased dramatically over the last three decades, particularly in Asia (Jennings et al. 2016), and currently represents 44% of the total global seafood production (Lopes et al. 2017). Farmed seafood has been shown to contain substantially lower Hg concentrations than taxonomically related wild-caught seafood (Karimi et al. 2012), providing a mechanism for reducing Hg exposure in some consumers. However, the context-dependent implications of these trends are uncertain for future trajectories of MeHg exposure to different human populations. In addition, responses of fisheries to global change drivers, as discussed in Domain 1, represent a key area of uncertainty that is likely to be extremely consequential for reducing human exposure to Hg.	

#### Artisanal small-scale gold mining (ASGM) and mercury mining

Socioeconomic trends can also influence human exposure to inorganic Hg, particularly from sources associated with ASGM. This mining is carried out in over 70 countries (mainly those with low- and middle-income economies) by approximately 10–15 million miners, including approximately 4–5 million women and children (UNEP 2013; WHO 2016). Within ASGM communities, Hg^0^ is used to amalgamate gold from processed ore, then the amalgam is subsequently heated to burn off the Hg as a vapor, leaving a concentrated gold ore. Annual Hg emissions from ASGM are estimated at 727 tonnes, making this the largest sector of Hg emissions, accounting for more than 35% of total global anthropogenic emissions of Hg (UNEP 2013). It has been argued that ASGM is a poverty-driven activity with pertinent micro- and macroeconomic drivers as well as push–pull factors (Hilson and Garforth 2012; Wilson et al. 2015). Biomonitoring surveys of ASGM workers and community members show that they have among the highest Hg exposures of any group worldwide (Gibb and O’Leary 2014; Basu et al. 2015b). This is not surprising considering the high amounts of Hg used in the practice, the limited use of personal protective equipment or Hg-free or -reduction techniques, and the overall rudimentary nature of most ASGM sites. The Hg exposure risks are further exacerbated by the fact that most ASGM operations are informal (and illegal in many countries) and thus sit outside regulatory and public health support programs (see Box 2).

**Table:** 

**Fig. 4:**
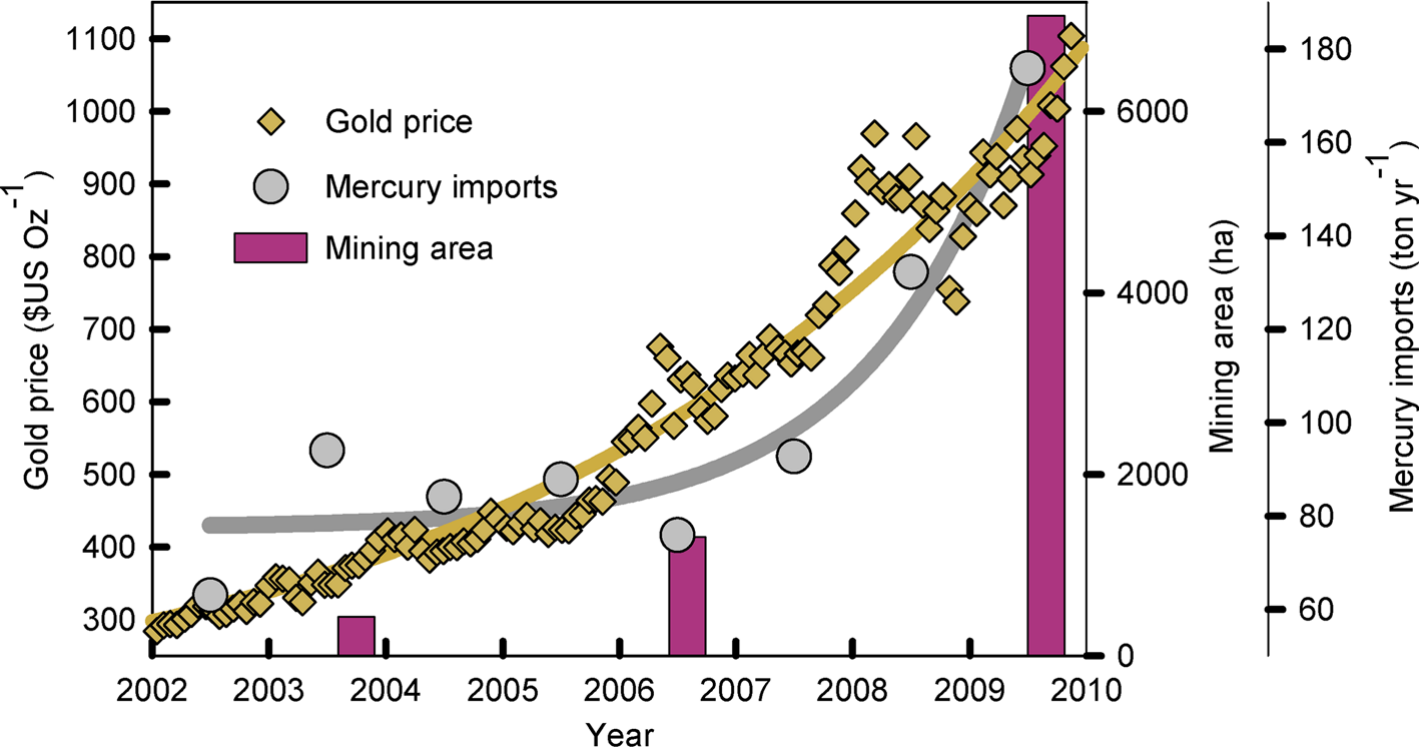
Relationship between international gold prices, Hg imports, and extent of ASGM mining are in Peru. Reprinted from Swenson et al. (2011)

**Box 2** ASGM as an example of an extrinsic global driver of human Hg exposure The prevalence and extent of ASGM is largely driven by local, community, and cultural factors, but global-scale drivers, such as economic market trends, are also important determinants. For example, sudden changes in the global price of gold correlated with the importation of Hg and the expansion of ASGM areas in Peru between 2003 and 2008 (Swenson et al. 2011). During this period, which included the global economic crash, the price of gold rose markedly and reported Hg imports into Peru increased by 42%. Satellite imagery showed that rates of forest conversion to mining increased 6-fold during this time period. Combining all these data, the authors reported strong correlations among these covariates as shown in Fig. 4, providing a striking illustration of the importance of global economics as a driver of Hg use and ecological degradation in ASGM regions. Similarly, ASGM gold production in Ghana increased 15-fold between 1995 and 2014 in response to a roughly 6-fold increase in the price of gold. During that time frame, the number of ASGM miners in Ghana rose 33-fold, from approximately 30,000 in 1995 to 1 million in 2010, and forest cover decreased from 32.7 to 21.7%. Although estimates of Hg imports are difficult to obtain, limited estimates indicate that they increased from 272 kg Hg in 2010, when approximately 21,000 kg of gold was produced, to 2015 kg of Hg imported in 2013 when more than 41,000 kg of gold was produced.	

#### Medical, personal care, and cultural use

Although fish consumption and ASGM are the dominant drivers of Hg exposure to most humans, other sources such as medical and personal care products can be important contributors for specific subsets of individuals. Historically, Hg was used for therapeutic medical purposes such as treatment for syphilis and as a component in teething powders (Clarkson and Magos 2006). These uses have been phased out, but modern medical applications still exist. In particular, Hg continues to be used as a component of dental amalgams and vaccines. These uses have generated controversy because of the known toxicity of Hg. The public health weight of evidence is somewhat equivocal, but generally has not pointed to adverse health outcomes associated with amalgams (Clarkson and Magos 2006) or vaccines (Stratton et al. 2001). However, developing biomarkers of Hg sensitivity and subtle adverse effects of Hg exposure (see Domain 3) may further inform this topic (Branco et al. 2017; Crowe et al. 2017; Dorea 2017; Modabbernia et al. 2017). Voluntary steps to reduce uses of Hg in medical applications have been taken by many producers and in many countries, but it still represents a significant source of Hg to many populations and thus should to be considered in cumulative exposure assessments.

Cultural norms, traditions, and religious ceremonies are additional extrinsic drivers that can influence Hg exposure (particularly to Hg^0^ and Hg^II^) in certain human populations. Application of Hg-containing skin-lightening creams to achieve desired physical appearances is a common practice in some populations, with prevalence rates as high as 50% in some countries (Dlova et al. 2015; Gbetoh and Amyot 2016; Lartey et al. 2017). Unregulated products often contain either Hg or calomel, or Hg is intentionally added by the user (Copan et al. 2015). Mercury exposure also occurs through certain ethno-medical and religious practices such as sprinkling elemental Hg on the floor of the home or burning it in candles to ward off evil spirits (Zayas and Ozuah 1996; Masur 2011). In 2005, a population-based study in New York, found that women from Dominican and Caribbean cultures had higher urine Hg levels than those from other cultural groups (McKelvey et al. 2011), and the increased concentrations correlated with both the use of Hg-containing skin-lightening creams (as mentioned previously) and the use of elemental Hg in religious and ethnic practices. Similarly, another study of prenatal exposures to Hg in a U.S. population of predominately Caribbean immigrants found that high maternal urine Hg levels were associated with religious use of Hg during pregnancy, including candle burning and religious charms, while elevated cord blood Hg was associated with fish consumption during pregnancy (Geer et al. 2012). Of note, these studies of individuals from cultures with specific religious uses of Hg were conducted in the U.S.; while public health resources are available to these U.S. immigrants in order to reduce risks from Hg exposure, the founder populations in other countries may not have the same level of access to those types of resources. Thus, these published Hg exposure risks may be significantly higher in other countries (Johnson 1999; Riley et al. 2001; Newby et al. 2006).

### Individual- and molecular-level drivers: Intrinsic

#### Genetics

Exposure biomarkers (e.g., blood, hair, or urine Hg concentrations) are important tools for monitoring and estimating Hg exposure in human populations. However, discrepancies exist in the relationships between actual exposure as measured through dietary intake or inhalation estimates and realized exposure as measured through biomarkers (Christensen et al. 2016; Awata et al. 2017; Branco et al. 2017), suggesting that there are other drivers that modify the pathway from actual to realized Hg exposure. Over the past decade research has shown that genetic and epigenetic factors may influence realized Hg exposure (reviewed by Basu et al. 2014; Llop et al. 2015). For example, using data from a group of 469 dental professionals, Basu et al. (2014) estimated Hg intake through self-reported seafood consumption surveys, measured hair Hg levels, and genotyped all participants to try and better understand whether genetic information could improve exposure assessments. As expected, estimated Hg intake and hair Hg levels were positively correlated, but there was an interaction between Hg intake and genotype such that the intake–biomarker relationships differed among genotypes. Specifically, among avid fish consumers (i.e., those who ate six cans of tuna per week), there was an 8-fold difference in average hair Hg levels (0.7–6.2 μg/g) among those with different polymorphisms in the *SEPP1* (rs7579) gene. Cross-sectional studies from across the world are also showing that polymorphisms in certain environmentally responsive genes (e.g., glutathione, metallothionein, and selenoenzyme families) are associated with the main effect (i.e., carriers of wildtype and variant genes have different Hg biomarker levels) and gene–environment interactions (i.e., exposure–biomarker relationships are different between carriers of the wildtype and variant genes). Similar observations linking genotype and realized Hg exposure have been made in studies of various populations, including dentists (Wang et al. 2011; Parajuli et al. 2016), students (Gundacker et al. 2007, 2009), Indiginous riverine populations (Barcelos et al. 2013, 2015), and gold miners (Custodio et al. 2005; Harari et al. 2012; Engstrom et al. 2013). Despite the expanding breadth of information on this topic, research to date has largely focused on adults and much less is known about the impact of genomic drivers on early-life Hg exposure situations or changes in Hg exposure across the life course. Further, most of the studies have focused on populations that are mainly exposed to elevated inorganic Hg sources, with MeHg exposures generally within background levels. Finally, there remain inconsistent findings across studies (see reviews by Basu et al. (2014) and Llop et al. 2015) which has limited the adoption of genetic factors into Hg risk assessment. If genetics plays a role in pharmacodynamic Hg routing and tissue distribution, then similar exposure profiles among individuals of different genotypes could result in large differences in biomarker concentrations. This not only influences the utility of biomarkers for estimating Hg risk, but may also be important for determining the sensitivity of individuals to Hg exposure. In addition to genetics, there is some emerging evidence from both animals (Pilsner et al. 2010; Basu et al. 2013) and humans (Hanna et al. 2012; Goodrich et al. 2013) to suggest that Hg is epigenetically active, and that Hg-induced methylation of certain genes is associated with either a main effect of Hg exposure or a gene–environment interaction.

#### Gastrointestinal factors

Despite the common assumption that most (> 95%) ingested MeHg is bioavailable, substantial evidence exists suggesting that this may not be valid. In particular, a review by Bradley et al. (2017) identified a range of MeHg bioavailability in seafood, between 12 and 79%, across 20 different studies. Factors such as type of seafood, cooking method, and the presence of certain nutrients all affected Hg bioavailability. More recently, because microbiota in the gut can affect the speciation of Hg prior to elimination, the impact of the gut microbiome has received attention for its role in the excretion of MeHg (Rothenberg et al. 2016). Thus, inaccuracies likely exist in estimates of exposure and risk that assume constant assimilation efficiencies of ingested MeHg.

**Fig. 5:**
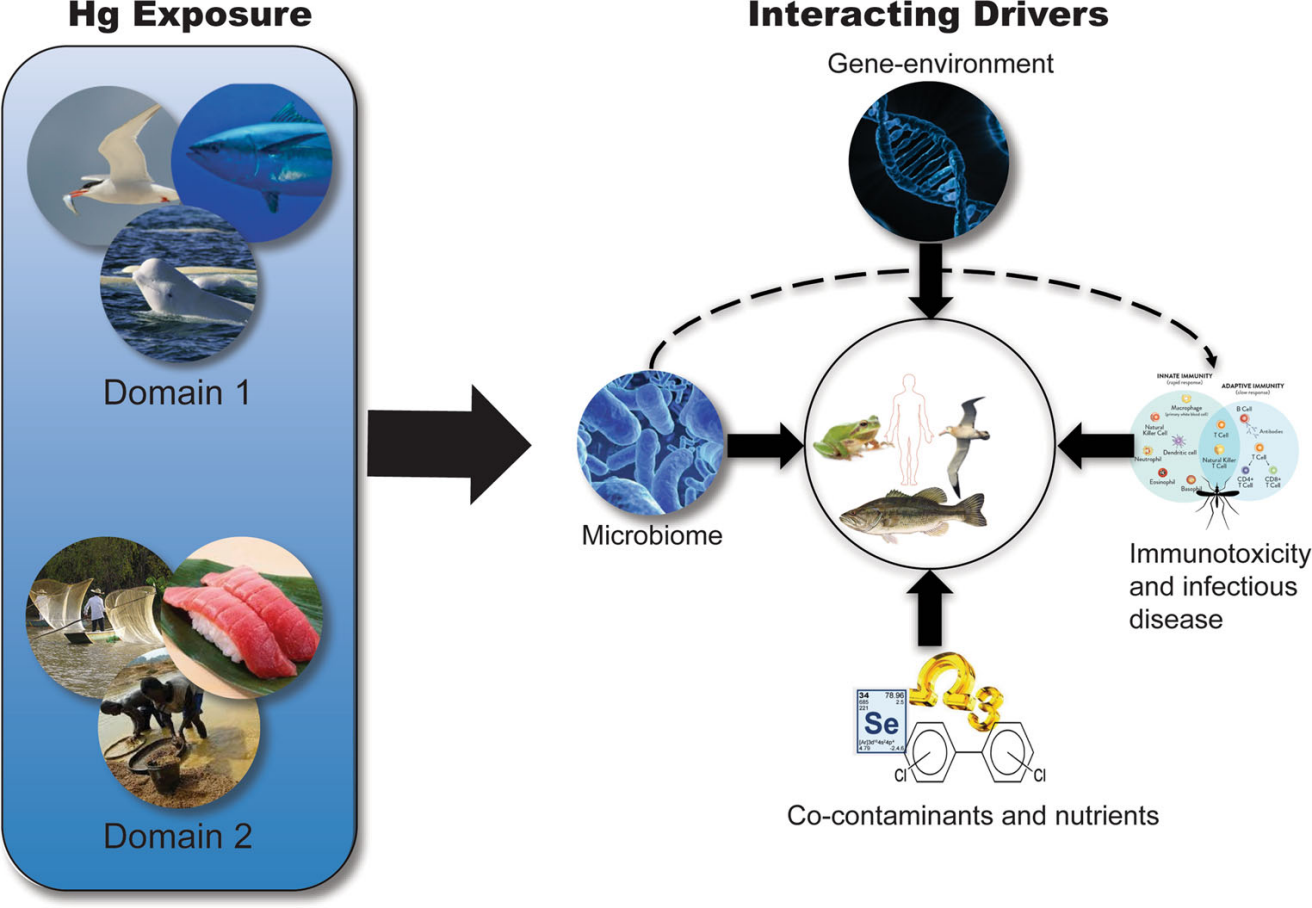
Conceptual model for Domain 3, evaluating interactions between Hg exposure and key extrinsic and intrinsic drivers on adverse health outcomes in humans and wildlife

## Domain 3: Interacting extrinsic and intrinsic drivers that influence the adverse health effects of MERCURY in humans and wildlife

Mercury exposure can affect multiple organ systems, and manifests as diverse adverse health outcomes in humans, fish, and wildlife. The devastating neurologic impacts of acute, high-level MeHg and elemental Hg exposure are well documented, but chronic, low-level exposure also has important health impacts that are less well understood. A number of recent reviews have summarized the range of outcomes, known mechanisms, and toxicity benchmarks associated with different levels of Hg exposure or in situ Hg concentrations in humans (Karagas et al. 2012; Sheehan et al. 2014; Ha et al. 2017), fish (Depew et al. 2012a; Wiener et al. 2012), and wildlife (Scheuhammer and Sandheinrich 2008; Depew et al. 2012b). Together, this comprehensive collection of reviews highlights both the pervasive occurrence of deleterious health effects associated with environmentally relevant Hg exposures, as well as the tremendous variability in the range of exposures that induce those effects. The variability in Hg sensitivity suggests that the onset of Hg toxicity is likely mediated by various interacting extrinsic and intrinsic factors. In this section, we examine potential interactions between Hg and other drivers that affect the processes, mechanisms, and health outcomes of Hg toxicity. These drivers represent a range of scales and, where applicable, we relate the drivers that modulate the health effects of Hg with the extrinsic drivers of Hg bioaccumulation and exposure described in Domains 1 and 2. Because toxicity of Hg is ultimately a physiological process, we do not separate human health outcomes from those of wildlife or other animals, but instead integrate examples across taxa where relevant.

We examine the interactions of Hg exposure with various drivers that modulate organism health, such as the microbiome, infectious disease, coexposures to other toxicants, diet and nutrition, and genetics (Fig. 5). These interactions are not exclusive to one another, but for simplicity, we evaluate each one independently. The true interactions are likely much more complex as multiple drivers are acting simultaneously. These drivers may (1) chemically modify Hg, as discussed in the “Microbiological factors: The microbiome and antibiotic resistance” section on *microbiological factors*; (2) modify associations between Hg exposure and disease, as covered in the “Immunotoxic effects of mercury and interactions with pathogens” section on *infectious disease*; (3) affect the risk of adverse health effects, as outlined in the “Nutrients and co-contaminants” section on *diet and co*-*contaminants*; or (4) independently increase the risks of Hg-associated health outcomes, as discussed in the “Genetics” section on *genetics*.

### Microbiological factors: The microbiome and antibiotic resistance

The microorganisms living commensally in and on humans and other animals exceed the number of somatic cells by a factor of 10 or more (Turnbaugh et al. 2007), and contain a number of genes that code for functions that support host physiology (Huttenhower et al. 2012). The aggregate genetic information encoded by these microbial communities is defined as the microbiome, and its recognition over the last decade has produced a paradigm shift regarding the role it plays in human and animal health (Kau et al. 2011; Muegge et al. 2011). The gut microbiome, in particular, is critical for immune function (Thaiss et al. 2016), nutrition (David et al. 2014), and xenobiotic metabolism (Dietert and Silbergeld 2015). Much is still unknown about the interactions between the microbiome and the health effects of Hg exposure, but emerging evidence suggests the presence of complex interactions that result in both positive and negative health outcomes for the host organism. For example, the gut microbiome can act as a co-factor that alters the toxicity of Hg by changing its speciation or sequestering Hg prior to absorption through biosorption and intracellular accumulation (Gadd 2010). The microbial genes responsible for Hg methylation have recently been found in both the vertebrate and invertebrate gut microbiome (Gilmour et al. 2013), and experimental studies have documented substantial Hg methylation within the digestive tracts of vertebrates (Rowland et al. 1975; Wang et al. 2013; Martin-Doimeadios et al. 2017). Moreover, emerging empirical evidence suggests that the gut microbiome also may demethylate Hg (Rowland et al. 1984; Wang et al. 2017), potentially reducing its toxicity.

The relationship between Hg, the gut microbiome, and host organisms extends beyond alterations of Hg toxicity in the host. Mercury sensitivity is common among microorganisms, and relatively low exposures may be lethal to many strains. Thus, Hg exposure can alter the gut microbiome itself, potentially affecting organism health (Madan et al. 2012). Mercury-induced changes to the microbiome can alter certain functional groups that are important for host physiology and health (Li et al. 2008). However, the scientific evidence regarding the extent and importance of these outcomes is only starting to be generated and significant research is still needed. Some of the risk of Hg toxicity to the microbiome is ameliorated by Hg-resistance genes that have evolved in many bacterial strains (Pal et al. 2015), and these genes can be exchanged among bacteria through horizontal gene transfer. Importantly, Hg-resistance genes are commonly associated and co-located with genes that also confer antibiotic resistance (Skurnik et al. 2010). Thus, Hg exposure can co-select for microbial communities in the gut that are resistant to both Hg and multiple antimicrobials (Li et al. 2017). The interaction between Hg and antimicrobial resistance may be a particularly significant impact of Hg on the health of biota.

Antibiotic resistance is among the most pressing public health threats (Levy and Marshall 2004), and progress managing it remains a significant challenge. The potential contribution of Hg exposure to this expanding crisis could have important implications for the role of Hg in global health. However, only limited evidence exists regarding the magnitude of Hg’s contribution to global antibiotic resistance. The correlation between Hg exposure and antibiotic resistance emerged in the 1990s with the observation that use of dental amalgams led to shifts in fecal bacterial communities, with a higher proportion of Hg-resistance genes present after amalgam use (Summers et al. 1993; Wireman et al. 1997). Resistance to antibiotics was substantially more prevalent in Hg-resistant strains of bacteria than those that were Hg-sensitive (Summers et al. 1993; Wireman et al. 1997). More recently, it was shown that the Wayampi Amerindians from French Guyana, who have high level of Hg exposure but limited access to antibiotics and little contact with the outside world, had a higher prevalence of antibiotic-resistant *E. coli* in fecal samples than Europeans with low Hg exposure and ready access to modern antibiotics (Skurnik et al. 2010). Another study of 2522 fully sequenced bacterial genomes and 4582 plasmids from a range of environments found substantial co-occurrence patterns between Hg-resistance and antibiotic-resistance genes (Pal et al. 2015). Moreover, antibiotic resistance was 2.5–10 times more prevalent in strains with varying expressions of Hg-resistance genes than those without Hg-resistance genes (Pal et al. 2015). That study also found that humans and domestic animals were among the host environments with the most prevalent co-occurrence of strains for both Hg resistance and antibiotic resistance. In addition, many of the strains that exhibited co-occurrence were of pathogenic clinical significance, such as *Escherichia*, *Staphylococcus*, *Salmonella*, and *Klebsiella*. Still, little is known about the prevalence of Hg- and antibiotic-resistance co-selection across different types of host taxa and its ubiquity in the environment. Recent work does suggest that co-selection extends to fish, where Hg exposure selected for gut microbiomes with up to 8-fold higher abundance of Hg-resistance genes, and resistance to multiple antibiotics was more common in Hg-resistant, as opposed to Hg-sensitive, bacterial colonies (Meredith et al. 2012; Lloyd et al. 2016).

Much more research is needed on the significance of this co-selection, but given the threat posed by antibacterial resistance to the health of humans and the environment, as well food security, it may have substantial, and as yet unquantified, implications regarding the health impacts of Hg exposure. Finally, the antibiotic resistance connection to Hg exposure may include important feedback loops leading to adverse effects of Hg toxicity, as both inorganic Hg and MeHg exposures can suppress immune function, increasing the likelihood of pathogenic infection (see “Immunotoxic effects of mercury and interactions with pathogens” section).

### Immunotoxic effects of mercury and interactions with pathogens

One of the major health threats associated with global change drivers such as invasive species and shifting climates is the potential emergence, resurgence, and redistribution of infectious disease (Wu et al. 2016). Because Hg also affects immune function (Sweet and Zelikoff 2001; Crowe et al. 2017), changes in infectious disease patterns may have a significant effect on human health outcomes. The immunotoxic effects of low level exposures to various Hg species are not well known, but within the last decade, a growing body of evidence in animal models (Nyland et al. 2012), wild animals (Fallacara et al. 2011; Lewis et al. 2013; Desforges et al. 2016; Becker et al. 2017), and humans (Sweet and Zelikoff 2001; Silbergeld et al. 2010) points to a range of immune system interactions with elemental, inorganic and MeHg exposures. Different Hg species induce different types of immunotoxic responses (Gardner et al. 2010a), and the mechanisms of immunotoxicity depend upon the magnitude of Hg exposure. Higher Hg exposures can affect the abundance of cells responsible for mounting an immune response (dendritic, B, and T cells), thereby impacting the majority of the production of cytokine signals. Conversely, lower-level Hg exposures only affect the production of cytokine signals, without changing immune cell counts (Silva et al. 2005b). This means that at lower Hg exposures, immune response can recover if cytokine-producing cells can return to basal function, whereas the death of immune cells at higher Hg exposures substantially inhibits immune response recovery. As in the nervous system, the immune system in many species, including humans, undergoes extensive pre- and postnatal development. Early exposures to Hg that are limited to in utero and early postnatal stages appear to reprogram the immune system, causing changes that persist into adulthood, long after exposure ceases (Silva et al. 2005a). Thus, Hg exposure can have long-lasting impacts on the ability of the immune system to respond stimulating events appropriately. The basic immune response is modified by these early exposures differently in males and females, and those changes affect the response to stimuli even into adulthood. Thus, males and females exposed to Hg early in life may have different risks in adulthood for autoimmunity and different responses to infection (Silva et al. 2005a).

The complexities of the interactions between immune function, disease, and Hg exposure are exemplified in experimental studies of the interactions between Hg and Coxsackievirus B3 infections. Early evidence indicated that viral infections altered the concentrations of Hg in target organs (Ilback et al. 2008), and the interaction between Hg exposure and disease response was dependent upon the relative timing of pathogen and Hg exposure. The virus alone could cause autoimmune myocarditis, whereas Hg alone had no myocarditic effect. However, when Hg exposure occurred prior to viral infection, the severity of autoimmune myocarditis was substantially increased, despite no changes in viral infectivity (Nyland et al. 2012). Conversely, Hg exposure after viral infection did not alter the severity of myocarditis compared to individuals who were not exposed to Hg (Nyland et al. 2012).

Despite experimental, correlational, and epidemiological evidence of Hg immunotoxicity, inconsistencies among studies, particularly those in human populations, have prevented rigorous risk assessments of the effects of Hg on immunological health. Some of these inconsistencies may be related to failure to account for interacting cofactors that modulate the Hg–immunotoxicology link. For example, in a sample population of 692 children from the U.S., there was limited evidence of a relationship between blood Hg concentrations and either measles or rubella antibodies, but after stratifying based upon key nutritional status indicators, the population with specific nutritional deficiencies (high methylmalonic acid (MMA), low folate, high homocysteine) exhibited a positive relationship between blood Hg concentrations and antibody titers for the two viruses (Gallagher et al. 2011, 2013). Other inconsistencies exist between indices of autoimmunity and Hg exposure. Specifically, increased risks for autoimmune dysfunction, as determined by elevated serum titers of autoantibodies such as anti-nuclear autoantibodies (ANA), have been demonstrated for certain Hg-exposed populations in South America compared to unexposed groups (Silva et al. 2004; Gardner et al. 2010b; Nyland et al. 2011). However, no evidence for such an association was found in Hg-exposed European and U.S. populations (Crowe et al. 2015; Monastero et al. 2017). Some work suggests that an interaction between Hg and malaria exposure may trigger Hg-induced autoimmunity in some populations but not others (see Box 3).

**Table:** 

**Box 3** Mercury and malaria The relationship between Hg and malaria represents a complex interaction between Hg and the immune system. Malaria acts as a global driver that may influence Hg toxicity, and Hg can influence the immune response to malaria infection. The human response to malaria infection involves several layers of immediate and chronic events in the life cycle of the parasite, as well as interactions between activation and suppression of immune response, all of which may be affected by Hg. Specifically, Hg exposure interacts with the human immune system to impair host resistance to the parasite (Silbergeld et al. 2000), increasing the likelihood of infection. Mercury also compromises the development of acquired immunity to malaria and may result in increased severity of the symptoms of infection (Silbergeld et al. 2000). In addition, Hg exposure that occurs subsequent to malaria infection induces autoimmune dysfunction. Epidemiological studies in areas with endemic malaria, coupled with exposures to Hg through ASGM and contaminated fish consumption found increased malaria infection rates compared to other populations (Dorea et al. 2003, 2005). In addition, biomarkers of autoimmune dysfunction were found to be common in Hg-exposed populations with a history of malaria contraction, whereas biomarkers were not detected in Hg-exposed populations with limited malaria exposure (Motts et al. 2014). Malaria-endemic areas of the world exhibit a substantial geographic overlap with major regions of ASGM. This creates a nexus for potential interactions between Hg and malaria exposure that can exacerbate the deleterious effects associated with these individual stressors. Evidence of these interactions exist from studies in Brazil (Duarte and Fontes 2002). Increases in gold prices stimulated expansion of ASGM activities throughout Amazonia, and physical activities associated with mining, coupled with an increase in human occupancy, resulted in a rapid increase in the incidence of malaria infections. Specifically, the alteration of stream habitats created new breeding habitats for *Anopheles* mosquito vectors of malaria (de Oliveira et al. 2013). In addition, much of the population attracted to gold mining in remote areas for work and economic gain were immunologically naïve in terms of exposure to malaria and thus potentially more likely to contract malaria (Doolan et al. 2009). As the workers moved back and forth between the mining areas and their places of origin, they transported malaria with them, increasing rates of infection outside Amazonia (Silbergeld et al. 2002). Thus, global socioeconomic drivers (gold prices) influenced human exposure to Hg in this region, which may have indirectly contributed to the spread of malaria to more populated regions. In addition, given the documented interactions between Hg and malaria exposure, it is likely that human health was additionally compromised. Cumulatively, this series of findings has significant public health implications and suggests that interactions between Hg and infectious diseases may be an understudied but highly relevant aspect of the risks to human and ecological health posed by Hg exposure. In addition, the distribution of many pathogens is currently being altered by climate change, which is facilitating the spread of their intermediate hosts (Wu et al. 2016). Indeed, models predict that under a range of forecasted climate scenarios, the distribution of malaria is likely to expand, particularly into higher elevations of tropical latitudes (Caminade et al. 2014). Further quantification of the health implications of the malaria–Hg interaction will facilitate estimates of future risks.	

### Nutrients and co-contaminants

Nutrients and other contaminants can have substantial ameliorative or exacerbating influences on Hg toxicity, complicating estimates of the health risks associated with Hg exposure (Rice 2008). Although there are potentially limitless combinations of coexposure mixtures that may influence health outcomes associated with Hg exposure, the most common include selenium (Se; (Cuvin-Aralar and Furness 1991; Hu et al. 2017), other biomagnifying contaminants such as polychlorinated biphenyls (PCBs) and other halogenated organic compounds (Braune et al. 2005; Burgess et al. 2005), co-occurring contaminants in point-source situations such as lead and arsenic in mining areas (Basu et al. 2011, 2015a), and dietary nutrients like omega-3 fatty acids (Gribble et al. 2016). Of these, Se is arguably the most compelling because of the extreme complexity and magnitude of its antagonistic interaction with Hg, which has evoked the greatest discussion and debate with no clear consensus. The antagonistic relationship between Hg and Se has generated a broad body of literature including numerous review articles (Cuvin-Aralar and Furness 1991; Luque-Garcia et al. 2013; Bjorklund et al. 2017). Both in vitro and in situ studies from field and laboratory settings have demonstrated reduced severity of inorganic Hg and MeHg toxicity in response to Se exposure, but the degree of amelioration is at least partially dependent upon the Se species used and its exposure route. Selenite and organic Se compounds (selenocysteine and selenomethionine) tend to be more protective than species such as selenate (Cuvin-Aralar and Furness 1991; Khan and Wang 2009; Dang and Wang 2011). Although a consensus is still lacking, the ameliorative effects of Se on Hg toxicity have been postulated to occur through a range of mechanisms, including reducing assimilation and facilitating elimination of MeHg (Bjerregaard et al. 2011; Bjerregaard and Christensen 2012; Li et al. 2012; Huang et al. 2013), rendering Hg biologically unavailable through covalent bonding between Hg and Se (Yang et al. 2008), demethylation of MeHg in the liver or other organs (Eagles-Smith et al. 2009; Khan and Wang 2009), or through supporting the glutathione antioxidant pathway (Sormo et al. 2011). Various degrees of empirical support exist for each of these mechanisms, highlighting both the complexity of the Hg-Se interaction as well as its ubiquity across physiological processes. One commonly referenced concept emerging from the body of work on Hg–Se interactions is the contention that Se:Hg molar ratios greater than 1 confer protection from manifestations of Hg toxicity, whereas molar ratios less than 1 indicate a lack of protection from Se. This hypothesis served as a foundational concept for a more recently posited mechanism for Hg toxicity—that Hg irreversibly binds with Se, thereby interrupting the synthesis of critical selenoenzymes, creating a Se deficiency syndrome that disrupts many aspects of an organism’s physiology (Ralston and Raymond 2010). According to this proposed mechanism, the amount of Se would need to exceed a Se:Hg molar ratio of 1 in either diet or tissues, so that it could confer a protective effect against MeHg toxicity. Although intriguing, some studies challenge the validity of this hypothesis, as discussed below.

Contemporary research continues to demonstrate a lack of clarity regarding whether Se provides universal protection for MeHg exposure, or is specific to only some mechanisms of action, such as neurotoxicity. For example, dietary selenocysteine did not reduce MeHg uptake, or enhance Hg elimination in captive mink (Evans et al. 2016). In addition, MeHg toxicity in harbor seal lymphocytes was not ameliorated by Se, even at Se:Hg molar ratios of up to 101 (Das et al. 2016), whereas similar studies in beluga whales indicated only limited protection from Se at the highest MeHg doses (Frouin et al. 2012). Perhaps most important in terms of deviations from the idea of Hg–Se antagonism is their interaction with respect to reproduction. Recent studies have documented deleterious synergistic relationships between Hg and Se to both bird (Heinz et al. 2012) and fish (Penglase et al. 2014) reproduction. Specifically, at elevated Se exposures, Hg appears to exacerbate Se-induced reproductive toxicity. Few studies have experimentally tested this relative to other Hg–Se outcomes, and it is unclear if these interactions are constrained to only egg-laying species, or if viviparous placental reproduction is subject to a similar synergism. Regardless, these experimental findings highlight the need to continue developing a quantitative understanding of the mechanistic role of Se across Hg toxicity endpoints, and suggest that application of the Se:Hg molar ratio hypothesis may not be a panacea for reducing Hg-associated health risks in humans and wildlife.

Other dietary nutrients may also offset the deleterious effects of MeHg exposure, although not as directly as occurs with Se. Interactions with omega-3 fatty acids, such as eicopentaenoic acid (EPA) and docosahexaenoic acid (DHA), are of particular interest because of their known health benefits and co-occurrence at high concentrations with Hg in many marine fish species (Mozaffarian and Rimm 2006; Gribble et al. 2016). The potential interactions with Hg have largely focused on neurodevelopmental and cardiovascular outcomes. In general, limited evidence exists for substantial antagonism of Hg by omega-3 fatty acids, although some studies have found that accounting for omega-3 fatty acid or fish intake can improve estimates of Hg impairment for certain populations, suggesting that these fatty acids might have an ameliorating effect (Oken et al. 2008; Choi et al. 2014). Others have found that when populations are stratified by their levels of omega-3 fatty acids, relationships between Hg exposure and adverse outcome biomarkers such as oxidative stress tend to be enhanced in those with low omega-3 levels, and weakened in those with high omega-3 levels (Karimi et al. 2016b). The specific mechanisms underlying any potential protective effects of nutrients on MeHg toxicity have not been fully elucidated (Ha et al. 2017). Since MeHg and these nutrients are often present in the same exposure sources and affect the same toxicity endpoints, quantifying their individual effects and interactions is challenging. Despite this, the benefits of fish consumption on health parameters is well known, and developing quantitative tools to better determine the risk–benefit coefficient is needed to facilitate recommendations for balancing potential health concerns about MeHg exposure against the benefits afforded by key nutrients found in seafood (Chapman and Chan 2000).

Whereas nutrients such as Se and omega-3 fatty acids can help offset the negative consequences of Hg exposure, coexposure to other contaminants can have the opposite effect. Of particular concern are other biomagnifying contaminants, such as PCBs and other organohalogenated compounds, that may co-accumulate with MeHg and affect the same physiological systems impaired by Hg. Similar to omega-3 fatty acids, many other bioaccumulative contaminants in the environment exhibit collinearity with MeHg exposure, which can confound the determination of the interactive effects on organism health. In vitro and in vivo laboratory studies testing responses to Hg and co-contaminant exposures alone and in combination suggest that PCBs in particular interact with MeHg exposure to elicit neurologic effects when isolated exposures do not (Bemis and Seegal 1999; Roegge et al. 2004; Fischer et al. 2008; Cauli et al. 2013). Similarly, epidemiological studies with individuals stratified by Hg or PCB exposure indicate potential interactive effects, such as PCB-associated neurologic deficits only in the highest tertile of Hg exposure (Grandjean et al. 2001), or negative associations between prenatal MeHg exposure and cognitive abilities only in those with elevated prenatal PCB exposure (Stewart et al. 2003). Although limited, recent field studies with wildlife suggest that MeHg exposure may also interact with other endocrine-disrupting compounds to influence hormone expression, disturbance response, and reproduction (Goutte et al. 2015; Tartu et al. 2015a, b). Similarly, there is emerging evidence that many ASGM sites are co-contaminated with lead, arsenic, and cadmium (Basu et al. 2015b; Tirima et al. 2016), and more work is needed to better understand the toxicologic implications of these coexposures. Given the abundance of studies measuring coexposure of other contaminants with Hg, it is surprising that so few have quantitatively tested for interactive effects. This is clearly a major data gap, and more accurate determinations of risk from Hg coexposure with nutrients and other contaminants, in both epidemiological and field-based studies on humans and wildlife, are needed.

### Genetics

Although external environmental factors can influence individual sensitivity to Hg exposure, genetic and epigenetic factors may influence Hg exposure–outcome associations through alterations in toxicokinetics and toxicodynamics. In “Genetics” section, we outlined how carriers of certain genetic polymorphisms can have altered Hg exposure biomarkers. Other researchers have shown that neurodevelopmental disorders (Mitchell 2011) and cardiovascular disease (Ehret et al. 2011), both of which are relevant in terms of Hg-associated health risks, have genetic underpinnings and more importantly, are driven by complex gene–environment interactions. There is a limited, though slowly growing, body of evidence demonstrating that Hg gene–environment interactions are an important feature to be considered. For example, several studies have reported that Hg-associated effects on atypical urinary porphyrin excretion, neurobehavioral tests, and visual-motor performance were modified by genetic polymorphisms (reviewed by Basu et al. (2014)). Another study reported that the offspring of mothers with *GSTM1* and *GSTT1* deletions (genes involved in glutathione function) and higher blood Hg levels had increased risk for low birth weight (Lee et al. 2010). Variation in responses to MeHg exposure among families suggests that susceptibility to Hg exposure may be heritable (Varian-Ramos et al. 2013). Importantly, recent work with zebrafish demonstrated transgenerational abnormalities in the neurobehavior of unexposed F2 generation fish, and that the proximate cause was sperm epimutations in Hg-exposed parents (Carvan et al. 2017). These findings suggest Hg exposure in wild populations could have multigenerational effects on fitness, even after exposure is reduced. The prevalence of heritable neurodeficits due to Hg exposure in previous generations is unknown, but these findings indicated that germ-line genes appear to be targets for MeHg genotoxicity.

## Summary and research needs

Persistent knowledge gaps that can exist between Hg releases, bioaccumulation, and exposure, and the risks of deleterious health impacts to humans and other animals can create substantial uncertainty regarding the efficacy of Hg-reduction and mitigation actions. Intrinsic biological factors and extrinsic ecological and socioeconomic factors are important modulators of the realized exposures to MeHg and inorganic Hg, as well as health impacts on Hg-exposed organisms. Therefore, as Hg-reduction and mitigation efforts proceed, a quantitative understanding is needed of how these extrinsic and intrinsic drivers interact on the global, individual, and molecular scales to influence the risk of Hg exposure, toxicity, and associated health effects. Incorporating additional metrics representing these factors into monitoring programs would facilitate tractable approaches to statistically account for their interactive effects on Hg-related risks. Specifically, understanding the global extrinsic drivers that affect the four mechanisms of MeHg bioaccumulation and biomagnification (primary productivity, habitat use, bioenergetics, and food web structure) will help partition out their influence on Hg concentrations in ecosystems. To do so requires refining the quantitative understanding of how each of the four underlying mechanisms MeHg bioaccumulation act, and in what contexts they are altered by global extrinsic drivers. Development of data application tools that facilitate incorporating these metrics into monitoring programs with a standardized approach is an important need, and would enable more robust comparisons of analogous temporal trends of Hg bioaccumulation among geographically diverse locations. 

The emergence of high-throughput genetic tools has opened new avenues for understanding (1) how intrinsic genetic factors influence assimilation of and sensitivity to Hg; (2) epigenetic responses to Hg exposure that can reveal specific mechanisms of action; and (3) interactions between the gut microbiome and Hg. These emerging areas represent potentially important contributions to the field for refining risk estimates based on genetic variation among populations, and for developing more effective biomarkers of Hg exposure. Substantial research in these areas is still needed for them to be applicable to public health and ecological conservation. Similarly, not enough is known about the interactions between Hg exposure, immunotoxicity, and infectious disease. Clearly the immune system is highly sensitive to both inorganic Hg and MeHg exposure, but extrapolating that sensitivity to the toxicologic implications at the whole organism scale is an important next step, particularly as it relates to infectious disease interactions. Finally, despite nearly five decades of research, and evidence from hundreds of scientific articles supporting Se as strong antagonist to Hg toxicity, there is still no consensus regarding the efficacy of Se in ameliorating different manifestations of Hg toxicity. Consequently, widely accepted tools for incorporating Se into health risk assessment for Hg exposure are still lacking. New criteria are emerging that take into account some of the mechanisms of the Hg–Se antagonism (Zhang et al. 2014; Ralston et al. 2016); however, extensive epidemiologic research is still needed to validate their safety for health risk assessments and their applicability to wildlife health. This is an important research direction that deserves continued focus. This paper demonstrates that the context within which Hg exposure occurs in the environment is a critical aspect that influences risk of exposure and adverse health outcomes. Future efforts to develop context-dependent assessments will facilitate improved risk estimates for humans and wildlife.
